# The Cross Talk between Cancer Stem Cells/Cancer Initiating Cells and Tumor Microenvironment: The Missing Piece of the Puzzle for the Efficient Targeting of these Cells with Immunotherapy

**DOI:** 10.1007/s12307-019-00233-1

**Published:** 2019-11-22

**Authors:** Shilpa Ravindran, Saad Rasool, Cristina Maccalli

**Affiliations:** Research Department, Sidra Medicine, Al Luqta Street, PO Box 26999, Doha, Qatar

**Keywords:** Cancer stem cells/Cancer initiating cells, Immunosurveillance, Adaptive immune responses, Innate immune responses, Tumor microenvironment, Immunotherapy

## Abstract

Cancer Stem Cells/Cancer Initiating Cells (CSCs/CICs) is a rare sub-population within a tumor that is responsible for tumor formation, progression and resistance to therapies. The interaction between CSCs/CICs and tumor microenvironment (TME) can sustain “stemness” properties and promote their survival and plasticity. This cross-talk is also pivotal in regulating and modulating CSC/CIC properties. This review will provide an overview of the mechanisms underlying the mutual interaction between CSCs/CICs and TME. Particular focus will be dedicated to the immunological profile of CSCs/CICs and its role in orchestrating cancer immunosurveillance. Moreover, the available immunotherapy strategies that can target CSCs/CICs and of their possible implementation will be discussed. Overall, the dissection of the mechanisms regulating the CSC/CIC-TME interaction is warranted to understand the plasticity and immunoregulatory properties of stem-like tumor cells and to achieve complete eradications of tumors through the optimization of immunotherapy.

## Introduction

Tumors are composed by heterogeneous cellular components including a rare subpopulation bearing “stemness properties” and being responsible of tumor initiation and progression. These cells have been denominated cancer stem cells (CSCs) or cancer initiating cells (CICs) [1–6]. CSCs/CICs share several characteristics with normal stem cells, such as the ability to self-renew and to give rise to differentiated progeny and the resistance to DNA damage-induced cell death [3, 5–12]. CSCs/CICs, through the cycling from proliferation to quiescence, expression of ABC drug pumps, high levels of anti-apoptotic proteins and resistance to DNA damage, are resistant to radiation and chemotherapy and play an important role in disease relapse and tumor progression [13, 14].

CSCs/CICs have been isolated from both hematological and solid tumors and they represent a rare subpopulation, comprising 0.01–10% of cells within the tumor [15]. They can be *ex vivo* identified based on their “stem cell-like” characteristics and the expression of certain cell surface and functional markers [16]. The identification of CSCs/CICs was first reported in leukemia, showing a hierarchical organization of tumor cells [17]. The leukemic cells were able to be engrafted upon transplantation of CD34^+^CD38^−^ cells into severe combined immune-deficient (SCID) mice, which eventually led to the identification of the hierarchical organization of tumors with few cells endowed with stemness and tumorigenic properties [17]. Since then, a variety of studies highlighted the existence of “stem-like” cancer cells in solid tumors with different histological origins [5, 18–23]. Multiple molecules (e.g., ALDH-1, CD133, CD44, CD24, CBX3, ABCA5, LGR5, etc) have been identified as CSC/CIC-associated markers with differential expression depending on the tissues of derivation, highlighting the high grade of heterogeneity of these cells [16] (Table 1). Most of these molecules are over-expressed by CSCs/CICs but are also shared with either differentiated tumor cells or normal stem cells [4, 34]. As a result, detecting the presence of these cells within tumor lesions though probing for CSC/CIC- associated markers has not provided conclusive results. The xenotransplantation in immune deficient mice represents a useful tool to demonstrate *in vivo* the tumorigenic properties CSCs/CICs [35]. Xenograft models have contributed to prove the existence within tumor lesions of cell population endowed with stemness properties that upon serial transplantation could propagate both tumorigenic CSCs/CICs and malignant cells with differentiated phenotype without tumorigenic properties [18]. These subpopulations can be identified only through transplantation in immune deficient mice [4, 36–38].

**Table 1:** Markers expressed by CSCs/CICs isolated from solid tumors and their role as TAAs

Marker^a^	Tumor type	Recognition by T cells^b^	Reference
ALDH1	CRC; breast and gastric cancer; melanoma	√	[24]
CD133	GBM, pancreas, lung, ovarian, prostate, and gastric cancer	√	[25–27]
CD44	CRC, head and neck cancer		
EpCAM	CRC, Retinoblastoma		[28]
EpCAM CD44 CD24	Pancreatic cancer		
CD24	CRC	√	[29, 30]
SOX2	GBM	√	[31]
CBX3	Ostocarcinoma		
LGR5	CRC		
ABCB5	Melanoma		
CD90	Liver cancer		
HSP DNAJB8	RCC	√	[32, 33]
CD166	CRC; NSCLC		

^a^Markers commonly identified as associated with CSCs/CICs; ^b^ the role of these antigens in eliciting T cell-mediated immune responses against CSCs/CICs

CRC: colorectal cancer; GBM: glioblastoma multiforme; NSCLC: non-small cell lung cancer; RCC: renal cell carcinoma

Nevertheless, the available CSC/CIC-associated markers are dependent on spatial and temporal features, with their modulation occurring in relation to their inoculation in immunodeficient mice, proving the high level of plasticity of these cells and that none of the available markers can be exploited to monitor the *in vivo* fate of these cells [1, 39, 40] CSCs/CICs, similarly to normal stem cells, require a “niche” to allow the survival of these cells and their cycling from quiescence to proliferation and to maintain stemness and multipotency [41–43]. The “niche” is represented by the tumor microenvironment (TME), which is composed of multicellular and dynamic compartments that include fibroblasts, endothelial, stromal, mesenchymal and immune cells [41]. The interaction of TME with stem-like cancer cells can regulate the fate of these cells through modulating the proliferation, differentiation, immunological properties and resistance to therapies [44–50].

**Fig. 1:**
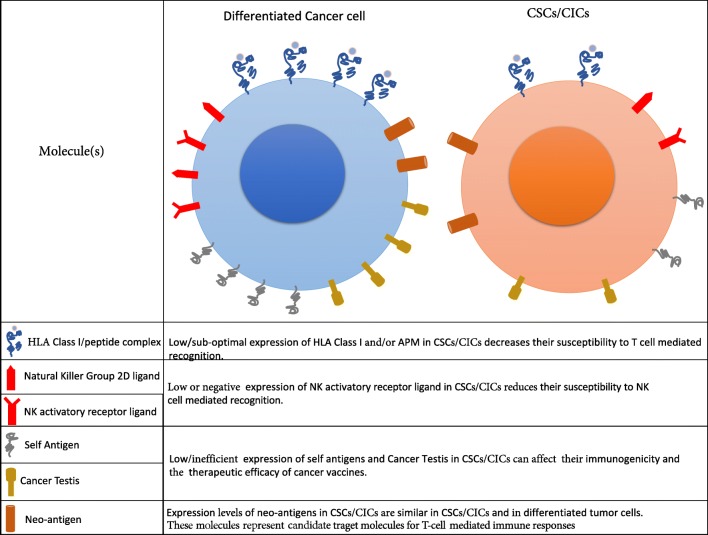
Differential immunogenic profile by CSCs/CICs vs. bulk tumor cells. CSCs/CICs can express defective levels of HLA molecules and APM components leading to low immunogenicity and escape from immune responses. In the presence of efficient expression of ligands of NK-associated activatory receptors, these cells can become susceptible to NK cell recognition. Moreover, TAAs can be expressed at suboptimal levels by CSCs/CICs. Neoantigens, generated by somatic mutation bearing tumor cells are equally expressed by both CSCs/CICs and differentiated tumor cells. The latest TAAs represent highly immunogenic target molecules, since they are not expressed by normal cells. APM: antigen processing machinery; CSCs/CICs: cancer stem cells/cancer initiating cells; NK: natural killer cells

**Fig. 2:**
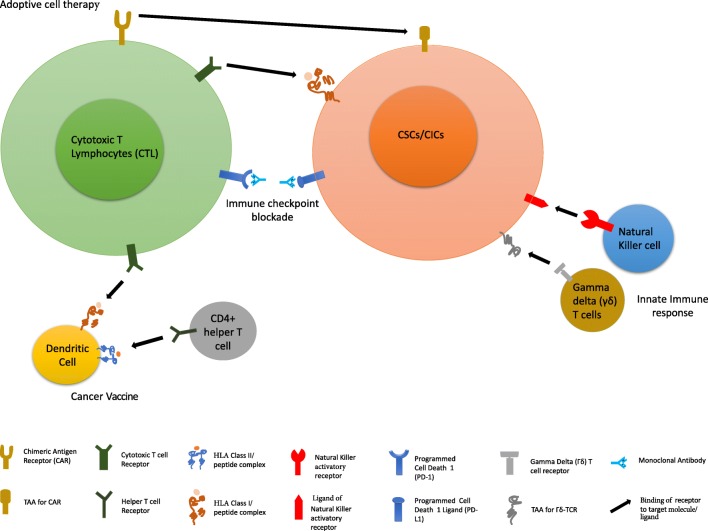
Immunotherapy strategies to target CSCs/CICs. An overview of immunotherapy approaches including adoptive cell therapy with either 1. TCR or CAR engineered T lymphocytes; 2. Immune check point blockade with mAbs; 3. Cancer vaccination with TAAs expressed by both CSCs/CICs and differentiated tumor cells; 4. Innate immune response or 5. γδ T cell recognition of tumor cells. Combination of either multiple immunotherapy approaches or with standard therapies warrant further investigation to assess the efficacy in increasing the immunogenicity of CSCs/CICs and to implement the targeting of these cells by immune responses. CSCs/CICs: cancer stem cells/cancer initiating cells; TAA: tumor associated antigen

The high grade of heterogeneity and plasticity of CSCs/CICs can depend on their tissue of derivation and, importantly, on their cross-talk with TME [4, 16, 51–53]. Limiting the isolation and the functional characterization of CSCs/CICs to the usage of phenotypic markers is unsatisfactory and do not consider the possibility that “stemness” function of tumor cells can be reversible, as shown by Quintana et al. for melanoma [1, 39]. Moreover, xenotransplantation of these cells in immune deficient mice is lacking the important variable of the TME and its role in affecting the fate of CSCs/CICs [1]. Therefore, the lack of standardized methods to isolate CSCs/CICs and of *in vivo* models allowing to monitor the cross-talk of these cells with TME can lead to the high extent of variability in assessing the functional properties of these cells and in preventing to accurately determine their fate and role in the tissue of origins and in the clinical outcome of cancer patients [54, 55]. The tool of sphere forming assay to propagate *in vitro* CSCs/CICs is too simplified, lacking the important component of TME and of the “niche”, preventing the constant monitoring of plasticity and heterogeneity of these cells (Figs. 1, 2).

Therefore, the combination of deep genomic, molecular and functional profiling of CSCs/CICs could represent a relevant method to achieve a comprehensive functional characterization of these cells and, possibly, of their role in tumor outcome [56].

CSCs/CICs have been identified as the tumor components responsible for resistance to standard therapy, as well as immunotherapy [10, 57–60]. Although clinical responses in cancer patients are observed following treatments, these cells can remain in the minimal residual disease and upon changes in the environment they can exit the quiescence status and give rise to novel malignant lesion(s) or even initiate the metastatic colonization [8, 61–63].

The extensive molecular and immunological characterization of CSCs/CICs is warranted in order to understand the mechanisms regulating their plasticity, quiescence, interaction with the TME and resistance to therapies and to immune responses.

## Immunological Profile of CSCs

### HLA Molecules and APM Components

The expression of HLA class I and class II molecules and APM has been investigated in CSCs/CICs isolated from colorectal cancer (CRC) and glioblastoma multiforme (GBM) showing an overall aberrant expression of these molecules, with, in some cases, failure in their modulation by the pre-treatment with IFNs (both alpha and gamma) or DNA demethylating agent (5-Aza CdR) [64, 65]. This impairment in antigen processing and presentation by CSCs/CICs lead, upon co-culture of these cells with autologous T cells, to a preferential selection and differentiation of TH2 type T cells and failure in eliciting effector functions [64, 65]. The suboptimal expression of HLA class I molecules and APM components was also reported in CSCs/CICs isolated from different type of solid tumors [64–69]. These peculiar observations suggested that the defective expression of HLA molecules could represent a tool for the identification of CSCs/CICs [70]. On the contrary, the side population (SP) cells derived from CRC and endowed with stemness properties, showed detectable level of HLA class I molecules as well susceptibility to antigen-specific cytotoxic T lymphocytes (CTLs) [71], however this study has been performed using long-term *in vitro* established cell lines, that could have lost phenotypic properties of primary CSCs/CICs. Indeed, stem-like cells isolated by sphere-forming assay displayed aberrant expression of HLA class I and APM components [16, 65]. Contradictory results were obtained also in glioblastoma multiforme (GBM); CSCs/CICs isolated as sphere forming cells from this tumor have been shown to exhibit the expression of HLA class I molecules [72] while, when applying these analyses to primary GBM-derived sphere forming cells, defective expression of HLA class I and APM molecules was detected [64]. Stem-like cells expressing ABCB5 and isolated from melanoma were found to express suboptimal levels of HLA class I molecules while they were positive for HLA class II (45). APM components (e.g., LMP2, LMP7 MECL-1, TAP1 and TAP2) detected through mRNA analyses were found to be expressed in tumor sphere-models from different solid malignancies, representing a tool for *in vitro* enrichment of stem-like cells and for the investigation of micro-metastasis [73]. However, HLA class I and class II molecules were down-modulated in these cells as compared to differentiated tumor cells, also following their pre-treatment *in vitro* with IFN-γ, highlighting an impairment of antigen presentation by these cells [64]. It needs to be considered that this study did not analyze the expression at protein levels of APM molecules, therefore post-transcriptional mechanism could affect their expression. Moreover, long term *in vitro* established cell lines were used to isolate tumor cell spheres while in other studies reporting defective expression of APM components, primary CSCs/CICs have been investigated. These examples highlight that an overall suboptimal immunogenic potency by CSCs/CICs resulting in low or impaired susceptibility to T cell mediated immune responses (Fig. 1). This represents a mechanisms of evasion by immune responses that is shared with normal stem cells, that could represent a typical feature of cells with stemness properties [74]. Importantly, the failure in the expression of HLA molecules by tumor cells was found as one of the mechanisms of failure of the clinical activity of immune checkpoint blockade agents in cancer patients [75, 76], indicating that either CSCs/CICs can display immune evasion mechanisms shared by differentiated tumor cells or that indeed the suboptimal expression of HLA molecules of these cells and their resistance to T cell recognition can protect these cells from immunotherapy interventions, leading to tumor recurrence or progression. However, the lack of standardization in methods for both, the isolation of cells with stemness properties and to analyze HLA and APM molecules, represents a limitation in providing conclusive results. Nevertheless, detailed analysis to identify the molecular mechanisms that lead to aberrant expression of HLA molecules and APM components are warranted.

The suboptimal expression of HLA class I molecules if associated with detectable NKG2D ligands, can drive the increased susceptibility of CSCs/CICs to Natural Killer (NK) cells. This phenomenon has been observed in CSCs/CICs from glioma, melanoma, and CRC [68, 77–79]. However, down-modulation of NKG2D ligands on CSCs/CICs has been documented, e.g., in GBM patients [64] suggesting that the expression of low levels of NK cell activating ligands can result in the impairment of anti-CSCs/CICs innate immune responses (Fig. 1). The expression profile of molecules activating innate immune responses on CSCs/CICs can be affected by their crosstalk with TME, and thus, by their plasticity that can influence the fate *in vivo* of these cells.

### Tumor Associated Antigens and Adaptive Immune Responses against CSCs/CICs

Tumor associated antigens (TAAs) can be recognized by T lymphocytes when exposed on the surface of tumor cells in the form of peptide/HLA complexes [80, 81]. They are categorized into three groups; (i) the overexpressed/self-antigens that are expressed at high levels by tumor cells and detectable, although at lower levels, on normal tissues (e.g., MART-1/Melan-A, hTERT, EGFR, survivin). (ii) Cancer testis (CT) antigens that are detectable on tumor cells and not on normal cells, except for testis and trophoblast (e.g., NY-ESO1, MAGE A3-A4, PRAME, CT83, SSX2). (iii) Neoantigens or mutated antigens derived by non-synonymous mutations in cancer cells (e.g., MUM-1, CDK4, ME1, ACTN4, HLA-A2) [82]. The neoantigens are higher immunogenic compared to differentiation/self TAAs since are tumor specific and do not induce tolerogenic mechanisms in immune cells [83–85]. Neoantigens have been shown to drive immune responses and to mediate efficient T cell recognition of tumor cells, leading to cancer eradication in patients treated with either mutanome based vaccines or adoptive cell therapy (ACT) with tumor infiltrating lymphocytes [84–86]. Notably, CSCs/CICs bearing a somatic mutation in the CRC-associated “driver” gene SMAD4, could elicit antigen-specific T cell responses directed to both stemness and differentiated components of tumor [87].

A transcriptome analysis of the SP cells and main population (MP) derived from CRC, breast and lung cancer revealed a preferential expression of 18 CT antigens (MAGEA2, MAGEA3, MAGEA4, MAGEA6, MAGEA12, MAGEB2, GAGE1, GAGE8, SPANXA1, SPANXB1, SPANXC, XAGE2, SPA17, BORIS, PLU-1, SGY-1, TEX15 and CT45A1) in CSCs/CICs [32]. The TAA DNAJB8, that is a member of the heat shock protein (HSP) 40 family, was found to be preferentially expressed in renal cell carcinoma (RCC); interestingly this protein played an important role in the maintenance of CSCs/CICs. DNAJB8-specific immune responses could be detected in a mice model study of DNA vaccination for RCC, rendering this molecule appealing for targeting CSCs/CICs by the immune system [32, 33] (Table 1). Recently a new antigen, Ankyrin repeat and SOCS box protein 4 (ASB4), was described as target molecule of CTLs recognizing CSCs/CICs and not the differentiated cellular components of the tumor [88]. Suboptimal expression of TAAs (MART-1, ML-IAP, NY-ESO-1, and MAGE-A) was reported in melanoma-derived CSCs/CICs (Fig. 1)[89]. Similar results were obtained in CSCs/CICs isolated from GBM and CRC (Fig. 1 and Table 1) [64, 65]. On the other hand, CD133^+^ CSCs/CICs isolated from melanoma were shown to express either NY-ESO-1 or DEAD/H (Asp-Glu-Ala-Asp/His) box polypeptide 3, X-linked (DDX3X) representing target of tumor-specific T cells [90, 91]. Other studies have described the isolation of T lymphocytes recognizing TAAs expressed by CSCs/CICs such as IL-13Rα2, SOX2 and CD133 in GBM, CEP55 and COA-1 in CRC and EpCAM in retinoblastoma (Table 1) [28, 65, 71, 92].

### Innate Immune Responses and their Relationship with CSCs/CICs

Natural killer (NK) cells are the first line of defense against cancer development and metastasis. NK cells have been described to efficiently recognize and kill *in vitro* CSCs/CICs isolated from CRC, melanoma and glioblastoma [68, 78, 93, 94]. The efficiency of NK cell-mediated lysis of CSCs/CICs was dependent on the expression of NCR ligands (NKp30 and NKp44), NKG2D ligands and when suboptimal or negative expression of HLA class I molecules were found on the surface of CSCs/CICs (Fig 1) [68, 78, 93–95]. Tallerico et al. found that CSC/CIC but not their differentiated counterpart of CRC is susceptible to NK cells [77]. Similar results have been reported in GBM and melanoma, highlighting that the amount of ligands of activatory NK receptors on CSCs/CICs was determinant for efficient innate immune responses [68, 77, 78]. In patients with acute myeloid leukemia (AML), the suboptimal expression of NKG2D ligands has been described as a mecahnisms of escape by tumor cells from NK cell recognition [96], confirming that these molecules can affect the susceptibility of cancer cells to innate responses. The observations that NKG2D ligands could represent as biomarkers for prediction of clinical responses to immune checkpoint blockade in melanoma highlight that the pattern of NKG2D ligands expression by tumor cells can affect the type and efficiency of elicited anti-tumor immune responses [97]. Therefore, the levels and pattern of expression of NKG2D ligands by tumor cells, including CSCs/CICs could be a predictive marker for the choice of the type of immunotherapy interventions.

Dendritic cells (DCs) are antigen presenting cells (APCs) that can activate either innate or adaptive immune responses [98]. In addition, they play an important role in the formation of anti-tumor T- and B cell immunologic memories [99]. Immature DCs can capture the tumor-derived antigens by phagocytosis or pinocytosis and then migrate to lymphoid organs where they present these TAAs in the form of HLA/peptide complexes to T cells, resulting in antigen-specific immune responses [100–102]. However, DCs depending on their morphological and phenotypic subtypes can either induce anti-tumor immune responses or promote tumor growth and progression [103]. The crosstalk of tumor with their TME is a crucial factor which results in the development of cancer [104]. Along this line, it has been described that high extent of expression of the chemokine (C-X-C motif) ligand 1 (CXCL1) by tumor and stromal cells can promote CSCs/CICs survival and proliferation and attract at tumor site DCs with suppressive functions, that could correlate tumor progression and poor survival of patients [104].

Macrophages represent important players for innate immune responses and can act as APCs similarly to DCs [105]. Based on their phenotype and functions they can be distinguished in two subpopulations: 1. The M1 subtype that are characterized by elevated pro-inflammatory cytokines, such as IL-12, IL-1*β*, IL-6, and tumor necrosis factor *α* (TNF-*α*), increased expression of HLA class II molecules, generation of reactive oxygen and nitrogen intermediates and ability to induce TH1-type T cell responses [106]. 2. In the presence IL-4, IL-10, and IL-13, macrophages can polarize towards M2 phenotype. These cells express scavenging, mannose and galactose receptors, IL-10, vascular endothelial growth factor (VEGF), matrix metalloproteinases (MMPs) and activation of the arginase pathway, leading to pro-tumoral effects [105–107, 109]. The cross-talk between CSCs/CICs and TAM is orchestrated by STAT3 signaling [102, 103]. Upon iper-modulation of STAT3 in TAM, they can promote stemness, survival and proliferation in cancer cells while the latest cells can induce the immunosuppressive properties of TAM, leading to the impairment of cancer immune-surveillance [110].

Myeloid derived suppressor cells (MDSCs) are immune cells endowed with suppressive functions that can inhibit the effector functions of immune responses [111]. The frequency of these cells either at tumor site or in the circulation has been described as a prognostic factor for patients’ survival as well as of responsiveness to immunotherapy [112]. Interestingly, STAT3 can lead the differentiation of monocytes towards MDSCs in pancreatic tumors [113] regulating also the development of CSCs/CICs [113]. The secretion of pro-inflammatory cytokines and chemokines by tumor cells can induce the differentiation and recruitment of immunosuppressive cells that can also contribute to sustain the inflammatory TME and to the interaction and reciprocal influence of CSCs/CICs and their niche [110, 113–115]. Another key regulator of the cross-talk between CSCs/CICs and TAM and DCs is represented by CD47 [116]. This molecule is over-expressed by CSCs/CICs of B cell malignancies. The binding of this molecule to the signal regulatory protein alpha (SIRPα), that mediates phagocytic functions in DCs and macrophages, has been shown to mediate the impairment of innate responses [116]. The cross-talk between CSCs/CICs and myeloid cells can affect both the fate and immunological profile of these cells, with implications for their susceptibilities to immune responses. Further studies should be designed to dissect the interactions of CSCs/CICs with different immune cells, although the major limitation is represented by the lack of *in vivo* models to monitor the interaction of these different immune cell population in the context of TME.

## Immunomodulatory Properties of CSCs/CICs

The immunological profiling of CSCs/CICs has revealed that they share some characteristics with embryonic, hematopoietic and mesenchymal stem cells displaying immunoregulatory functions that render these cells invisible to immune responses and able to escape from tumor immune responses [4, 74, 117]. The principle mechanisms governing the immunomodulation of CSCs/CICs are described below.

### Cytokines, Chemokines, Growth Factors & Immune Checkpoint Molecules

The observations that CSCs/CICs isolated from different tumor types can secrete soluble factors, such as Galectin-3, GDF-15, IL-10, IL-13, PGE2 and TGFb, or express immune checkpoint molecules with immunosuppressive functions, have suggested that these cells can regulate the impairment of immune responses as well as regulating a pro-tumoral TME (Table 2) [4, 51, 18–130]. These immunosuppressive factors have been described to induce the differentiation of regulatory T cells (Tregs) or MDSCs and M2 macrophages, resulting in the impairment of effector functions of innate and adaptive responses [4, 51, 130, 131]. Moreover, pro-inflammatory cytokines such as IL-6, IL-8, IL-10 and IL-13, released by CSCs/CICs can contribute to maintain an inflammatory and suppressive TME representing the “niche” sustaining cellular stemness (Table 2) [132, 133]. Indoleamine 2,3-dioxigenase (IDO), that mediates the catabolism of tryptophan, has been shown to be expressed by CSCs/CICs, contributing to the differentiation of Tregs, skewing the cytokine profiling of T cells toward TH2 -type and inhibiting the survival and proliferation of CTL (Table 2) [133, 134].

**Table 2:** Immunomodulatory molecules detected in CSCs/CICs

Molecule	Function^a^	Activity in CSCs/CICs^b^	Reference
IL-4	Cytokine involved in differentiation of naïve T cells to Th2.	Inhibition of TH1 cell mediated immune responses.	[65, 118]
IL-10, IL-13	Anti-inflammatory cytokines	Suppression of CTL functions; differentiation of Tregs and MDSCs	[89, 119]
TGFB	Growth factor with potent inhibitory function	Tregs differentiation, inhibition of TH1 responses	[89, 120]
STAT3	Transcription factor with a potential anti-inflammatory function	Maintenance and proliferation of CSCs/CICs; differentiation of MDSCs, iDCs, M2	[115, 121]
GDF15	Growth and differentiation factor related to cellular stress.	Inhibition of anti-tumor immune responses	[122]
Galectin-3	Protein with important role in cell-cell adhesion and interactions with the extracellular environment.	Inhibitor of T cell mediated immune responses	[89, 123]
IDO	Enzyme involved in tryptophan catabolism	Suppression of TH1 type immune responses and differentiation of Tregs.	[34, 51, 64, 65, 123]
CD200	A glycoprotein that regulates myeloid cell activity and inhibits macrophage lineages.	Immune suppression and regulation of anti-tumor activity.	[124, 125]
PD-L1	Ligand of PD-1 and Immune checkpoint molecule	Inhibition of CTL immune responses.	[64, 65, 126, 127]
B7-H3 and B7-H4	Immune checkpoint molecules	Immunomodulation of cellular immune responses	[34, 64, 65, 128, 129]

^a^: function of the molecules listed in the Table

^b^: Activity of these molecules when expressed by CSCs/CICs

iDC: suppressive dendritic cell; MDSC: Myeloid derived suppressive cells; M2: M2 phenotype of monocytes/macrophages; TH1: T helper type 1; TH2: T helper type 2

In addition, the CSC/CIC-associated expression of IL-4 and CD200, through cell-to-cell interaction, lead to the inhibition of T cell effector functions (Table 2) [65, 135]. It has been demonstrated that the over-expression of IL-4 by CRC-derived CSCs/CICs has led to inefficient TCR-mediated proliferation and antigen recognition of CTLs [65]. Interestingly, the neutralization of this cytokine by mAb could overcome the T cell mediated anti-tumor impairment and induce antigen-specific recognition of both CSCs/CICs and differentiated tumor cells [65]. This study showed that, upon up-regulation of HLA class I and APM expression through IFN-γ treatment of CSCs/CICs, T cells could specifically recognize a neoantigen, SMAD4, generated by a non-synonymous mutation bearing stem-like cells and bulk tumor cells [65]. Thus, CTL reactivity against CSCs/CICs and the TAA-specific immunosurveillance could be improved by the usage of strategies to correct the low immunogenic profile of these cells.

The immune suppressive profile of CSCs/CICs has been also confirmed by evidences describing the expression by these cells of immune checkpoint molecules (e.g. CTLA-4, PD-L1, B7-H3 or B7-H4) (Table 2) [4, 34, 64, 65, 136]. These observations highlighted the similarities between CSCs/CICs and normal stem cells in terms of the immune profile [34, 137, 138]. Moreover, altered expression of STAT3 pathway in CSCs/CICs can also affect their immune suppressive activity through inhibiting T cell proliferation and activation, inducing the differentiation of Tregs and triggering T cell apoptosis [121]. The observations reported above show that multiple mechanisms and molecular pathways are either up-regulated or aberrantly activated in CSCs/CICs resulting in their immune suppressive properties, therefore the blockade of these signaling through the combination of inhibitory agents should be considered in order to rescue the tumor-specific immune responses.

### MicroRNAs

miRNAs are non-coding RNAs regulating at post-transcriptional levels, through complementary binding to target mRNA, the expression of genes [139]. The altered regulation of gene expression in tumor cells can occur by both up- or down regulation of miRNA [139]. The most common activity of miRNAs in CSCs/CICs is represented by the control of the expression of either oncogenes (e.g., MiR34a, MiR31 or MiR205) or tumor suppressor genes [139]. The aberrant expression of few miRNAs, such as miRNAs 451and 199b-5p, has been shown to affect stem-like cell properties isolated from different type of tumors (e.g., GBM, breast cancer and medulloblastoma) [139–143]. Of note, miRNAs displaying regulatory activity on immune-related genes (e.g, miRNA-199a that can regulate the IFN-mediated responses) can play a role in the differentiation of mammalian CSCs/CICs [144]. The level of miRNA-124, through regulating the expression of STAT3, can affect the efficiency of anti-CSC/CIC T cell responses in GBM [145]. Along this line, miR203 and miR92 can control the stemness and immunological profiles of melanoma cells [146, 147].

## Immune Evasion and Tumor Dormancy

Tumor dormancy is represented by quiescent cells that can remain occult and undetectable by regular diagnostic methods for long intervals of time, even after initial clinical responses to therapies [148]. Quiescence of cells is the ability to exit cell cycle and remain in G0 phase until permissive environmental condition will lead to enter back into the cycling phase. This is considered one of the principle mechanisms underlying tumor dormancy. CSCs/CICs display the ability to cycle between quiescence and proliferation and together with their resistance to therapies represent the link between these cells and tumor dormancy [2, 5, 8, 9, 149–151]. Furthermore, the immune suppressive mechanisms associated with CSCs/CICs can orchestrate the evasion of these cells from immune recognition and immunosurveillance, and could be considered additional factors responsible of tumor dormancy [152].

An important mechanisms of immune-surveillance is the homing of immune cells to the tumor site, which ultimately form the immune infiltrate. Tumors arising from epithelial breast cancer are known to possess high levels MHC class I molecules and of infiltrating T effector cells and M1 macrophages. The immune infiltrate from mesenchymal like breast cancer tumors exhibit low levels of MHC class I molecules, high levels of PD-L1 and contain Tregs, M2 like macrophages as well as exhausted T cells [153]. CSCs/CICs that are considered the architects of their own microenvironment [154], as well as generated by epithelial-to-mesenchymal transition (EMT) can be potentially responsible for the type of immune infiltration depending of their pattern of immune profile.

Common gene expression patterns have been found in normal mammary stem cells and dormant tumor cells from breast cancer suggesting the possible presence of stem-like cells in dormant tumors [151]. In addition, different cell sub-populations could be isolated from relapsed AML endowed with differential tumorigenic ability depending on their up-regulation of stemness signaling [155].

A better understand of the relationship between stemness properties, immunological profile of CSCs/CICs and tumor dormancy will provide insights on the mechanisms of therapeutic resistance of these cells and will allow to identify strategies for complete tumor eradication.

## Immunological Targeting of CSCs/CICs

### Cancer Vaccines

The recognition of TAAs expressed by CSCs/CICs by T cells have been documented (see Table 1). These *in vitro* or *in vivo* models were based on the usage of TAAs that represented sources of antigens for the therapeutic administration of cancer vaccines in cancer patients [81]. However, the principle limiting factor of the clinical efficacy of this strategy is represented by the usage of “self”/tolerogenic TAAs, shared with normal tissues [81]. The low or negative expression of these categories of antigens and of CT-TAAs by CSCs/CICs can represent an additional reason of failure of high rate and long duration of clinical responses observed in cancer patients treated with cancer vaccines [4, 34]. In addition, the sub-optimal levels of HLA class I molecules and APM by stem-like cells can drive the failure in targeting CSCs/CICs by cancer vaccines leading to the development of tumor dormancy and tumor recurrence, although the observance of initial clinical efficacy of these therapeutic interventions [4, 34].

DC-based vaccines, exploiting these cells as APC to present TAAs to T cell-mediated responses, represent also a therapeutic strategy for cancer patients showing encouraging clinical activity [102, 156–162]. DCs loaded with either CSC/CIC-lysates or mRNA isolated from these cells represented source of antigens for vaccination in the context of Phase I/II clinical trials of GBM patients [163, 164]. These studies provided proof of principle of improved overall survival of cancer patients treated with CSC/CIC targeted immunotherapy [163–165]. Immune responses, with, in some cases increased frequency of circulating NK cells, were detected in patients showing clinical benefit from these treatments [163, 165]. Of note, these therapeutic interventions could overcome the failure of CSCs/CICs in expressing efficient levels of HLA class I molecules and in presenting TAAs to T cells, documenting for the first time, that cancer vaccine, if eliciting NK cell-mediated responses, could target stem-like tumor cells [158, 163–165]. Tumor cell clones expressing immunogenic neoantigens can undergo immune selection due to the recognition and elimination by T lymphocytes, leading to the survival of tumor cell clones not expressing strong immunogenic antigens and maintaining the expression of low immunogenic TAAs [166] (and see https://www.biorxiv.org/content/10.1101/536433v1). This process is also associated with immune evasions mechanisms developed by both tumor cells and TME [166].

In some tumors, the decrease in antigen presentation is a result of epigenetic silencing of the genes involved in antigen presentation machinery. The usage of demethylating agents such as 5-Aza-2′-deoxycytidine to reduce the methylation of genes involved in antigen presentation, is a potential strategy to increase the antigen presentation in these CSC/CICs. The effect of demethylation has been shown in CSCs/CICs from breast cancer, where it resulted in high expression levels of TAP1, which is involved in antigen presentation [167].

In addition, increased antigen presentation also improves the potential of discovering novel antigens, which can then be helpful in development of new anti-cancer vaccines (Fig. 2).

### Immune Checkpoint Blockade

Immune checkpoints, including CTLA-4, PD-1 and PD-L1, are important physiological regulators of innate and adaptive immune responses [168]. Biological inhibitory agents have been clinically developed, revealing striking therapeutically success [169–175]. However, a significant proportion of cancer patients failed to benefit from these therapies.

The effectiveness of immune checkpoint blockade (ICB) is largely dependent on the tumor microenvironment [176]. Tumors such as melanoma, bladder cancer and non-small cell lung cancer (NSCLC) can be characterized as “hot” tumors due to their inflamed TME, high levels of and neo-antigen expression and of T cell infiltration and detection of PD-L1. These tumors have been reported to be associated with higher frequency of susceptibility to immune checkpoint treatments. On the other hand, prostate cancer is considered to be a “cold” tumor, due to minimal level of T cell infiltration, and limited response to single agent checkpoint inhibition [176].

Expression of immune checkpoint molecules has been observed in CSCs/CICs from different histological origins [51]. PD-L1 expression was detected at high levels in CSCs/CICs isolated from primary human head and neck squamous cell carcinoma (HNSCC), gastric and breast cancer, CRC and GBM [34, 64, 65, 177–179]. ,CSCs/CICs could theoretically be targeted *in vivo* by immune checkpoint blockade agents, enhancing the clinical efficacy of cancer vaccines, as demonstrated in a mouse model [180]. However, recent reports describing that clinical failure of these therapies was associated with defective expression of HLA class I molecules by tumor cells [75, 76], suggest that these cells might evade from the ICB-mediated unleash of immune responses (Fig. 2).

### Adoptive Cell Therapy

ACT is represented by the isolation of T lymphocytes from cancer patients, their *ex vivo* expansion, and the infusion back into patients [181–183]. In addition, T lymphocytes engineered to express TCR with high affinity for a cognate TAA could be exploited for ACT studies [183]. Highly encouraging and sustained responses mediated by adoptively transferred TCR targeting the TAA NY-ESO-1 have recently been reported in different tumor types, such as breast cancer and myeloma [86, 184]. Nevertheless, the antigen choice is highly relevant to prevent severe toxicities due to “off-target” cross-reaction with normal tissues sharing the same antigens or expressing molecules mimicking the TAAs [182].

Neoantigens have been described as candidate TAAs efficiently recognized by T cells that can be exploited for ACT of cancer patients and, interestingly, CTL targeting these antigens could be isolated from tumor infiltrating lymphocytes (TILs) of melanoma and other type of malignant lesions [183, 185, 186]. Nevertheless, ACT to target neoantigens can represent a promising approach for treatment of cancer patients upon assessment of HLA expression by both CSCs/CICs and differentiated tumor cells and, in case of suboptimal levels of expression, the achievement of their up-regulation by pre-treatment with immunomodulating agents [4, 34, 51].

T cell can be genetically modified to express a chimeric antigen receptor (CAR) that is composed of epitope-specific domains isolated from mAbs linked to T cell-derived activatory/costimulatory molecules [182, 187–189]. CAR-T cells can recognize TAAs independently on the expression of HLA molecules and APM components [189]. CAR-T cell therapy for some subgroups of hematological malignancies represent the salvage intervention leading to stable clinical responses and improved overall survival of patients refractory to standard therapies or with recurrences [187, 189–192]. The usage of CAR-T cell therapy for solid tumors is currently under investigation, showing encouraging results in cancer patients with aggressive tumor types, including malignant mesothelioma, pancreatic cancer and GBM [193–197].

CAR-T cells targeting TAAs, such as CD133, EGFRvIII, EpCAM, CSPG4 and B7-H3, expressed by different type of solid tumors, including CSC/CIC components, have been developed in pre-clinical studies [196–201]. These studies have shown that the targeting of TAAs that are expressed only by tumor cells, including CSCs/CICs, and not by normal cells, could provide the rational for safe and efficient clinical development of CAR-T cells therapy for these tumors(Fig. 2) [200, 202]. Moreover, the combination of CAR-T cells targeting dual TAAs, EGFRvIII and CD133, has been used for the successful therapeutic treatment of a patient with advanced cholangiocarcinoma [203].

CAR-T cells targeting NKG2D ligands on CSCs/CICs have been investigated and tested both *in vitro* and *in vivo* [204, 205]. CSCs/CICs from glioblastoma expressing detectable NKG2D ligands could be efficiently targeted by CAR- T cells. These tools showed to efficintly eliminate also xenograft tumors [205]. Nevertheless, a major limitation associated with the use of these CAR-T cells is represented by the variability of the levels of NKG2D ligands on the surface of CSCs/CICs, depending on their origin and methodology used for their *ex vivo* isolation.

Nevertheless, further investigations aimed at a comprehensive genomic and immunological characterization CSCs/CICs are warranted to implement the efficiency and safety of ACT strategies.

## Conclusions

Recent advances in the genomic, molecular and immunological profiling of CSCs/CICs have contributed to the identification of dysregulated molecular pathways that orchestrate stem-like cancer cells and their interaction with TME. The heterogeneity and plasticity of these cells and the mutual effect of TME and CSCs/CICs on the resulting anti-tumoral or pro-tumoral environment, represent the principle limitations in predicting the fate of these cells and their role in cancer patients’ outcome. In addition, these cells have been identified as key players in therapeutic resistance of tumors and in the development of their dormancy. The down-modulation of HLA molecules and NK activatory ligands on CSCs/CICs, through decreasing their susceptibility to T or NK cell targeting, might represent one of the principle factors leading to resistance to immunotherapy. Although, the mechanisms regulating the levels of HLA molecules and NKG2D ligands on CSCs/CICs are yet to be dissected. Due to the complexity of the cross-talk between CSCs/CICs and TME and the high plasticity of these cells, it is difficult to predict what type of immune cells could play a relevant role in targeting CSCs/CICs. Multifactorial investigations, including the immunological profile, immunomodulating molecules, the interaction with TME and the type of immune cell infiltration will allow to provide insights. Nevertheless, the available tools to isolate and characterize CSCs/CICs, such as spheroids, immunodeficient mice, antigenic profile, are unsatisfactory to dissect the cross-talk of these cells with TME. Moreover, the development of pre-clinical *in vivo* models engrafted with human immune system is desirable to allow the monitoring of the interaction of CSCs/CICs with TME.

The targeting of CSCs/CICs by immunotherapy could result in the complete tumor eradication and stable clinical responses in cancer patients. This goal could be achieved by the design of combination of strategies based on innate and/or antigen-specific T cell responses with immunoregulatory agents that can render CSCs/CICs susceptible to cell-mediated immunosurveillance. In cases where epigenetic factors are responsible for low antigen presentation, the usage of demethylating agents could represent a potential strategy to overcome the low expression of HLA molecules.

Molecular approaches dissecting the fate of CSCs/CICs within tumor tissues will allow to develop immune-based precision medicine approaches and to identify biomarkers predictive of patients’ responsiveness to therapies.

## Abbreviations

ALDH: Aldehyde dehydrogenase
APC: Antigen presenting cells
APM: Antigen processing machinery
CAR: Chimeric antigen receptor
CIC: Cancer initiating cell
CRC: Colorectal cancer
CT: Cancer testis
CTLA-4: Cytotoxic lymphocyte antigen-4
CSPG4: Chondroitin sulphate protidoglycan 4
HLA: Human leukocyte antigen
IDO: Indoleamine 2,3-dioxygenase
GBM: Glioblastoma multiforme
GDF-15: Growth differentiation factor-15
IFN: Interferon
IL-4: Interleukin 4
IL-10: Interleukin 10
IL-13: Interleukin 13
IL-13α2: α2 chain of IL-13 receptor
mAb: Monoclonal antibody;
MDSC: Myeloid derived suppressor cell
NSCLC: Non-small cell lung cancer
PD-1: Programmed death 1
PD-L1: Programmed death ligand 1
RCC: Renal cell carcinoma
STAT3: Signal transducer and activator of transcription 3
TGFB: Transforming growth factor beta
TAA: Tumor associated antigen
Treg: T regulatory cell.
